# A population pharmacokinetic study of ampicillin therapy in hospitalized foals

**DOI:** 10.1093/jvimsj/aalag021

**Published:** 2026-02-23

**Authors:** Anisa Bardhi, Julien Scala-Bertola, Ronette Gehring, Jole Mariella, Francesca Freccero, Raffaele Scarpellini, Carolina Castagnetti, Zoubir Djerada, Andrea Barbarossa

**Affiliations:** Department of Veterinary Medical Sciences, University of Bologna, Via Tolara di Sopra 50, Ozzano Emilia I-40064, Bologna, Italy; Laboratoire de Pharmacologie, CHU de Reims, UR 3801 Pharmacologie et Pathologies Fragisilisantes, Université de Reims Champagne-Ardenne, 51 Rue Cognacq-Jay, Reims F-51000, France; Service de Pharmacologie Clinique et Toxicologie, CHRU-Nancy, Université de Lorraine, Rue du Morvan, Vandœuvre-Lès-Nancy F-54511, France; IMoPA, Université de Lorraine, CNRS, 09 Avenue de la Forêt de Haye, BP 20199, Vandœuvre-Lès-Nancy F-54505, France; Division of Veterinary Pharmacotherapy and Pharmacy, Department of Population Health Sciences, Faculty of Veterinary Medicine, Utrecht University, Yalelaan 104-106, 3584 CM Utrecht, the Netherlands; Department of Veterinary Medical Sciences, University of Bologna, Via Tolara di Sopra 50, Ozzano Emilia I-40064, Bologna, Italy; Department of Veterinary Medical Sciences, University of Bologna, Via Tolara di Sopra 50, Ozzano Emilia I-40064, Bologna, Italy; Department of Veterinary Medical Sciences, University of Bologna, Via Tolara di Sopra 50, Ozzano Emilia I-40064, Bologna, Italy; Department of Veterinary Medical Sciences, University of Bologna, Via Tolara di Sopra 50, Ozzano Emilia I-40064, Bologna, Italy; Health Sciences and Technologies - Interdepartmental Centre for Industrial Research (CIRI-SDV), University of Bologna, Via Tolara di Sopra 50, Ozzano dell’Emilia I-40064, Bologna, Italy; Laboratoire de Pharmacologie, CHU de Reims, UR 3801 Pharmacologie et Pathologies Fragisilisantes, Université de Reims Champagne-Ardenne, 51 Rue Cognacq-Jay, Reims F-51000, France; Service Pharmacologie-Toxicologie, Pôle de Biologie Territoriale, Centre Hospitalier Universitaire de Reims, Rue du Général Koenig, Reims F-51100, France; Department of Veterinary Medical Sciences, University of Bologna, Via Tolara di Sopra 50, Ozzano Emilia I-40064, Bologna, Italy; Health Sciences and Technologies - Interdepartmental Centre for Industrial Research (CIRI-SDV), University of Bologna, Via Tolara di Sopra 50, Ozzano dell’Emilia I-40064, Bologna, Italy

**Keywords:** therapeutic drug monitoring, data modeling, dosage simulations, equine, antibiotics

## Abstract

**Background:**

Pharmacokinetic studies on ampicillin in foals are limited, underscoring a relevant gap in knowledge, particularly regarding the treatment of critically ill neonatal foals.

**Hypothesis/Objectives:**

To evaluate the adequacy of the ampicillin dosing regimen in hospitalized foals and, if necessary, suggest alternative dosing strategies to achieve effective ampicillin concentrations.

**Animals:**

Data were collected from 12 hospitalized foals.

**Methods:**

Foals were treated with 20 mg/kg ampicillin intravenously every 6 h. Plasma samples were obtained within the first 48 h, and the minimum inhibitory concentration (MIC) was determined for pathogen-positive patients. Ampicillin concentrations were quantified using liquid chromatography–tandem mass spectrometry. A population pharmacokinetic model was developed using a nonlinear mixed-effects approach (stochastic approximation expectation–maximization or SAEM algorithm), and a pharmacodynamic evaluation of different dose regimens was conducted using Monte Carlo simulations.

**Results:**

A 2-compartment model with first-order elimination was selected. Age significantly influenced peripheral compartment volume and clearance. The model demonstrated excellent internal validation, with 97% of observed values within prediction intervals and robust stability, as confirmed by bootstrap and visual predictive checks. Pharmacodynamic simulations indicated that a dosage regimen of 20 mg/kg every 6 h achieved optimal PTA (≥90%) for MICs of 0.06-0.25 mg/L with a 50% fT > MIC target. For higher MICs or a 100% fT > MIC target, more frequent dosing (q4h) and higher doses (30-40 mg/kg) were necessary.

**Conclusions and clinical importance:**

The results from the simulations highlight the clinical importance of adjusting dosing regimens based on foal characteristics and MIC to ensure effective treatment, especially in critically ill foals.

## Introduction

Foals are born immunocompetent but immunologically “naive,” relying on colostrum for passive immunity. Consequently, newborn foals are more vulnerable to a wide range of both pathogenic and nonpathogenic bacteria, predisposing them to infection and sepsis.^1^ Due to the difficulty of ruling out sepsis in compromised neonatal foals, and the serious risk to the health of the foal of delaying or withholding antimicrobial therapy in case of infection, prophylactic antimicrobial treatment is usually recommended.^2^ Sepsis represents one of the most relevant causes of morbidity and death in neonatal foals, with early and aggressive treatment critical for improvement in outcomes.^3^ Empirical treatment typically involves intravenous administration of broad-spectrum antibiotics, most often an aminoglycoside (gentamycin or amikacin^3–5^) combined with a beta-lactam.^4^^,^^6^^,^^7^ Ampicillin is a time-dependent antibiotic within the latter category, with a commonly recommended dosage of 20 mg/kg every 6 h^4^ as a first-line treatment for neonatal sepsis, with higher dosages (50-100 mg/kg q6h) suggested in severely ill foals.^8^

For beta-lactams to be effective, plasma concentrations must exceed the minimum inhibitory concentration (MIC) by at least 50% of the dosing interval in Gram-positive infections and 80% in Gram-negative infections.^6^^,^^9^^,^^10^ Understanding the optimal dosing regimens and therapeutic efficacy of ampicillin is crucial for its effective use in clinical settings, especially since age-related physiological differences, such as changes in liver and renal function, gastrointestinal absorption, and plasma protein binding, complicate predictions of drug efficacy in foals.^11–14^ In addition, due to the effects of diseases on drug metabolism and elimination, outcomes of pharmacokinetics (PK) and pharmacodynamics (PD) studies on antibiotics can provide useful information for adapting treatment protocols in severely ill foals.^11–14^ In this context, PK/PD modeling is a valuable tool, as it enables the prediction of the effects of a drug based on its concentration over time and its relationship to the MIC of the pathogen. These models can guide the establishment of the most effective dosing regimens, ensuring that therapeutic concentrations of ampicillin are sustained while minimizing the risk of toxic or adverse effects.^15–18^

Despite the increasing use of PK/PD modeling to optimize antibiotic therapy in veterinary patients,^15^^,^^19–22^ a significant gap remains in its application to foals falling ill during their first days of life, where standard dosing regimens often fail to account for their unique metabolic characteristics. The aim of this study was to develop a population pharmacokinetic (PopPK) model of intravenous ampicillin administration in a limited cohort of sick neonatal foals incorporating relevant physiological covariates such as age, body weight, serum albumin, and creatinine concentrations. Based on this model, Monte Carlo simulations were conducted to estimate the probability of target attainment (PTA) according to established PK/PD indices in this cohort.

## Materials and methods

### Data collection

Twelve neonatal foals (between 9 and 194.5 h of life) hospitalized and receiving ampicillin therapy (Vetamplius, Fatro S.p.A., Italy; 20 mg/kg every 6 h IV) were enrolled in this experiment. The ampicillin preparation used was a sodium ampicillin; the dose was calculated based on the ampicillin base content, in accordance with the information reported in the summary of product characteristics^23^ (10 g of ampicillin/50 mL of the final solution). Ampicillin was administered via a long-term jugular catheter (Long-Term MILACATH Single Lumen—16 Ga × 15 cm, MILA International, Inc., USA). The enrolled foals were diagnosed with various critical conditions, including perinatal asphyxia syndrome, uroperitoneum, prematurity/dysmaturity, neonatal isoerythrolysis, total failure of transfer of passive immunity, and omphalitis. Foals received standard-of-care neonatal treatments on individual basis during the study, and all survived. Blood samples were drawn from the jugular catheter with the push-pull technique^24^ and collected in EDTA tubes (S-Monovette EDTA K3E, 1.2 mL, Sarstedt AG & Co. KG, Germany) at multiple timepoints during the first 48 h of treatment (see Table S1), centrifuged at 2000 × *g* for 15 min to separate plasma, and subsequently stored at −80°C. Serum albumin and creatinine concentrations were measured by an automated analyzer (Beckman Coulter AU480, Ireland) before stating treatment for all enrolled foals, and the MIC values were determined for samples that tested positive for bacterial pathogens using the broth microdilution method according to the Clinical and Laboratory Standards Institution (CLSI) guidelines,^25^^,^^26^ using customized broth microdilution panels. Plasma samples were analyzed according to the ultra-high performance liquid chromatography coupled to tandem mass spectrometry (UHPLC–MS/MS) method described by Bardhi et al. (2025).^27^ Briefly, 10 μL of amoxicillin-d₄ internal standard solution was mixed with 100 μL of plasma. After adding 200 μL of acetonitrile, the samples were vortex-mixed for 30 s and centrifuged at 21 000 × *g* for 10 min. A 10 μL aliquot of the resulting supernatant was then diluted with 990 μL of 0.1% aqueous formic acid and injected into the UHPLC–MS/MS system (Waters, Milford, MA, USA). The method was linear for ampicillin concentrations ranging from 0.3 to 100.0 mg/L. Coefficients of variation for inter- and intra-assay precision and accuracy were < 13%.

The study protocol was approved by the Animal Welfare Committee of the University of Bologna (Protocol No. 358467, ID No. 4626).

### Pharmacokinetic analysis

Population PK modeling of ampicillin was performed using Monolix software (version 2024R1; Lixoft, Antony, France, http://lixoft.com/) using a nonlinear mixed-effects modeling approach implementing the stochastic approximation expectation–maximization (SAEM) algorithm.^28–30^ Concentrations below the lower limit of quantification (LLOQ) of the analytical method were treated as censored observations and were assumed to lie between 0 and the LLOQ. Pharmacokinetic analyses were performed on the original ampicillin concentrations. Log transformation was not applied prior to model fitting. After developing the population PK model with Monolix software, we used Simulx software (version 2024R1; Lixoft, Antony, France; http://lixoft.com/) for model-based simulations of candidate dosing regimens to be tested.

#### Basic model building

One-, 2-, and 3-compartment intravenous bolus models with no delay in administration and first-order elimination were initially tested.^31^^,^^32^ All individual parameters were assumed to be log-normally distributed. The between-subject variability (BSV) of PK parameters was described by an exponential model as θ*_i_* = θ_TV_ × exp(η_(θ,*i*)_), where θ*_i_* is the estimated individual parameter, θ_TV_ is the typical value of the parameter, and η_(θ,*i*)_ is the random effect for the *i*th patient. The θ*_i_* values were normally distributed with a mean of 0 and a variance ω,^2^ where ω was the standard deviation estimated by Monolix. The variance–covariance matrix of random effects was assumed to be diagonal. Correlations between the random effects were also tested. The log-likelihood estimation was performed with 10 000 Monte Carlo importance sampling. Finally, the most appropriate model was selected using the corrected Bayesian information criterion (BICc) and the relative standard errors (RSEs). Several error models were tested to describe the residual variability: a constant error model (*C*_obs_ = *C*_pred_ + *a* × *ɛ*), a proportional error model (*C*_obs_ = *C*_pred_ + *b* × *C*_pred_ × *ɛ*), and combined error models such as *C*_obs_ = *C*_pred_ + (*a* + *b* × *C*_pred_) × *ɛ* (combined 1) or *C*_obs_ = *C*_pred_ + (*a*^2^ + *b*^2^ × *C*^2^_pred_)^1/2^ × *ɛ* (combined 2), with *C*_obs_ is the observed concentration; *C*_pred_ the predicted concentration, *a* the additive residual error constant, *b* the proportional residual error constant, and *ɛ* the random error term.^26^

#### Covariates analysis

From the basic model without covariates, the effect of age, total body weight (TBW), serum albumin, and serum creatinine (Scr) as covariates on ampicillin PK parameters was tested. The parameter–covariate relationships were included as follows:


$$ {\mathrm{\theta}}_i={\mathrm{\theta}}_{\mathrm{TV}}\times \exp \left(\mathrm{\beta} \times{\mathrm{COV}}_i\right)\times \exp \left({\mathrm{\eta}}_{\left(\mathrm{\theta}, i\right)}\right) $$

with *β* the covariate effect to be estimated, COV*_i_* the covariate value for subject *i*, and η_(θ,*i*)_ is the random effect for the *i*th patient.

As described,^33–35^ each covariate was entered into the model one at a time. The covariate was retained if there was a decrease in BICc and BSV, a significant Wald test (*P* < .05) and a 95% CI estimated by bootstrapping (*n* = 1000), omitting the zero value.^33^

#### Model internal evaluation

Evaluation of the model was based on goodness-of-fit plots, as observations vs individual and population predictions, individual weighted residuals (IWRES) vs individual predictions and time, plots of normalized prediction distribution error (NPDE) vs population predictions and time.^36^ The visual predictive checks were performed (*n* = 1000).^33^ This plot shows the time course of the 10th, 50th, and 90th percentiles of the observed concentration profiles compared to the median and prediction intervals obtained from the simulated data. The 95% CI of all model parameters were estimated using bootstrapping method (*n* = 1000).^33^ Finally, shrinkage was determined based on the empirical SD of the random effects (sd(ηi)) and the estimated standard deviation (ω) from the population model. These values were computed using samples from the conditional distributions of each individual, according to the following formula: η-sh = 1 − sd(ηi)/ω.

#### Evaluation of different dose regimens using Monte Carlo simulation

Using the estimated distribution of ampicillin PK parameters in the final IV PK model (including residual error, BSV, and accounting for uncertainty in random and fixed effects), Monte Carlo simulations were performed using the Simulx software (version 2024R1; Lixoft, Antony, France, http://lixoft.com/) to generate 1000 PK profiles of ampicillin for each candidate regimen. Population parameters were drawn using the covariance matrix of the estimates previously estimated in Monolix. Ampicillin unbound (free) plasma concentration-time profiles were simulated using an average protein binding value of 15% reported in the literature.^37–39^ The PTA, defined as the percentage of time that the free drug remains above the MIC, was assessed by determining the percentage of simulated patients achieving a given PK/PD target (50% fT > 1 × MIC, 100% fT > 1 × MIC, and 50% fT > 4 × MIC). Dosing regimens that achieved PTA ≥ 90% were considered optimal.^40^ The range of MIC values chosen was within the range of MIC values commonly reported in the literature from 0.01 to 2 mg/L.^38^^,^^39^^,^^41^

## Results

### Development of the pharmacokinetic model for ampicillin

#### Cohort characteristics

A total of 205 plasma samples, 12 serum creatinine and serum albumin were analyzed from 12 foals enrolled in this study. The cohort characteristics for age, total body weight, serum albumin, and serum creatinine are shown in Table 1. The median number of samples taken from each foal was 13.^12–24^ One plasma sample with a very high aberrant concentration due to probable sample contamination was excluded from the analysis. In addition, 17 samples with concentrations below the LLOQ of the analytical method were treated as censored observations and assumed to lie between 0 and the LLOQ. Raw empirical observations, population predictions, and individual predictions are reported in Table S3.

**Table 1:** Cohort characteristics. Data are given as median and interquartile ranges.

Variables	Foals (*n* = 12)
**Age (h)**	78.0 [19.7-180.0]
**Sex**	Male (*n* = 6)
	Female (*n* = 6)
**Breed**	Standardbred (*n* = 6)
	Mixed breed (*n* = 1)
	Quarter horse (*n* = 3)
	Arabian horse (*n* = 2)
**Number of samples per foal**	13.0 [12.0-24.0]
**Total body weight (kg)**	48.5 [38.0-60.0]
**Serum albumin (g/L)**	28.9 [26.5-30.7]
**Serum creatinine (mg/L)**	12.8 [9.5-14.7]
**Diagnosis**	Omphalitis (*n* = 5)
	Dysmaturity (*n* = 1)
	Neonatal isoerythrolysis (*n* = 1)
	Umbilical cord hematoma (*n* = 1)
	Total failure of passive transfer and flexural deformity (*n* = 1)
	Perinatal asphyxia syndrome (PAS) (*n* = 1)
	PAS and prematurity (*n* = 1)
	PAS and uroperitoneum (*n* = 1)

#### Population pharmacokinetic modeling of ampicillin plasma concentrations

A population PK (PopPK) model was developed using plasma ampicillin concentrations obtained from the study cohort of hospitalized foals. The final selected model was a 2-compartment intravenous model with first-order elimination. Its BIC value was 1217.28, representing a decrease compared to earlier model versions (Table S2). Indeed, a 1- or 3-compartment intravenous bolus model and first-order elimination was also tested but did not improve the fit. These models resulted in higher BICc values of 1280.38 and 1232.47, respectively. In the initial 2-compartment model, the interindividual variability of the intercompartmental clearance (ωQ) and the typical value of the peripheral compartment volume (V2) exhibited high uncertainty, with RSE values of 74.8% and 76.6%, respectively. Both exceeded the predefined threshold of 50%. As a first step, ωQ was fixed at 0.49, which led to a decrease of the BICc value of −2.67 (BICc = 1214.61). In a second step, since age explained 155% of the variability of V2, and V2 was positively associated with log(Age) (β__V2_logAge_ = 1.38) (Table 2). Log(Age) was therefore added as a covariate to the volume V2 (*P* value of Wald test = 1.07 × 10^−2^). The new value of V2 was then fixed at 22.12 L resulting in a new decrease of the BICc value of −12.4 (BICc = 1204.88). In addition, age also explained 24% of the variability in Cl and Cl was positively associated with log(Age) (β__Cl_logAge_ = 0.40) (Table 2). As a final step, log(Age) was then added as a covariate to the clearance Cl (*P* value of Wald test = 2.56 × 10^−2^) resulting in a BICc decrease of −14.88 (BICc = 1202.40).

**Table 2:** Population pharmacokinetic parameters. Rse-sa: Relative se_sa estimated using recommended stochastic approximation. The population parameters were modeled as follows: log(Cl) = log(Cl_pop_) + β__Cl_logAge_ × log(age/61.8) + eta_Cl_; log(V1) = log(V1_pop_) + eta_V1_; log(Q) = log(Q_pop_) + eta_Q_; log(V2) = log(V2_pop_) + β__V2_logAge_ × log(age/61.8) + eta_V2_ with 61.8 years is the weighted mean of age in the population. For each parameter, eta is the random effect. ^*^: *P* value of Wald test = 1.07 × 10^−2^; ^**^: *P* value of Wald test = 2.56 × 10^−2^; ^**#**^: Population parameter estimates and their 95% CIs (2.5%-97.5% percentile) obtained by stochastic approximation using the Fisher Information Matrix. ^##^: Population parameter estimates and their 95% confidence intervals (2.5%–97.5% percentiles) obtained from a parametric bootstrap (*n* = 1,000) using the stochastic simulation and estimation (SSE) method, based on data simulated from the final model. Censoring limits were applied to the simulated datasets, defined between the lower limit of quantification (LLOQ) and 0 mg/L. Bias, expressed as a percentage, is the difference between the mean of the bootstrap estimates and the reference value, and is calculated as follows: (mean − reference)/reference^*^100.

Parameter	Value^#^ (rse-sa %)	95% CI value^#^	Median value^##^	95% CI value^##^	Bias (%)
	**Fixed effects**				
**Cl_pop_ (L.h^−1^)**	17.43 (17.74)	[12.41-24.48]	17.41	[11.92-24.97]	1.50
β__Cl_logAge_^*^	0.40 (39.19)	[0.092-0.7]	0.39	[0.027-0.70]	−2.47
**V1_pop_ (L)**	18.83 (11.21)	[15.15-23.41]	18.58	[14.34-23.10]	−1.07
**Q_pop_ (L.h^−1^)**	16.79 (32.31)	[9.30-30.34]	17.11	[7.94-50.80]	17.51
**V2_pop_ (L)**	22.12 (Fixed)	–	–	–	–
β__V2_logAge_^**^	1.38 (44.8)	[0.17-2.59]	1.33	[0.22-3.24]	3.18
	**SD of the random effects**				
**ωCl**	0.55 (25.18)	[0.34-0.88]	0.47	[0.22-0.76]	−14.13
**ωV1**	0.32 (32.50)	[0.18-0.58]	0.28	[0.082-0.48]	−13.02
**ωQ**	0.49 (Fixed)	–	–	–	–
**ωV2**	1.04 (39.53)	[0.51-2.09]	0.79	[0.15-2.29]	−14.89
	**Error model parameters**				
**a**	0.12 (36.18)	[0.063-0.23]	0.11	[0.0024-0.28]	−0.091
**b**	0.40 (8.30)	[0.34-0.47]	0.39	[0.34-0.46]	−0.61

Cl_pop_, V1_pop_ V2_pop_, and Q_pop_ are the typical values of the clearance of ampicillin, the volumes of the central and peripheral compartments, and the intercompartmental clearance, respectively. These population parameters were modeled as follows: log(Cl) = log(Clpop) + β_Cl_logAge × log(Age/61.8) + etaCl; log(V1) = log(V1pop) + etaV1; log(Q) = log(Qpop) + etaQ; log(V2) = log(V2pop) + β_V2_logAge × log(Age/61.8) + etaV2 with 61.8 years is the weighted mean of age in the population. β__Cl_logAge_ and β__V2_log_Age_ are the covariate effects of log(Age) on clearance (Cl) and of log(Age) on peripheral compartment volume (V2), respectively; ωCl, ωV1, ωV2, and ωQ represent the SDs of the interindividual variability (eta) associated with the typical population parameters Cl_pop_, V1_pop_ V2_pop_, and Q_pop_, respectively; a and b are the additive and proportional error coefficients of the combined 1 residual error model: *C*_obs_ = *C*_pred_ + (*a* + *b* × *C*_pred_) × *ɛ*, where *C*_obs_ is the observed concentration, *C*_pred_ is the predicted concentration, and *ɛ* is the random error term.

**Figure 1:**
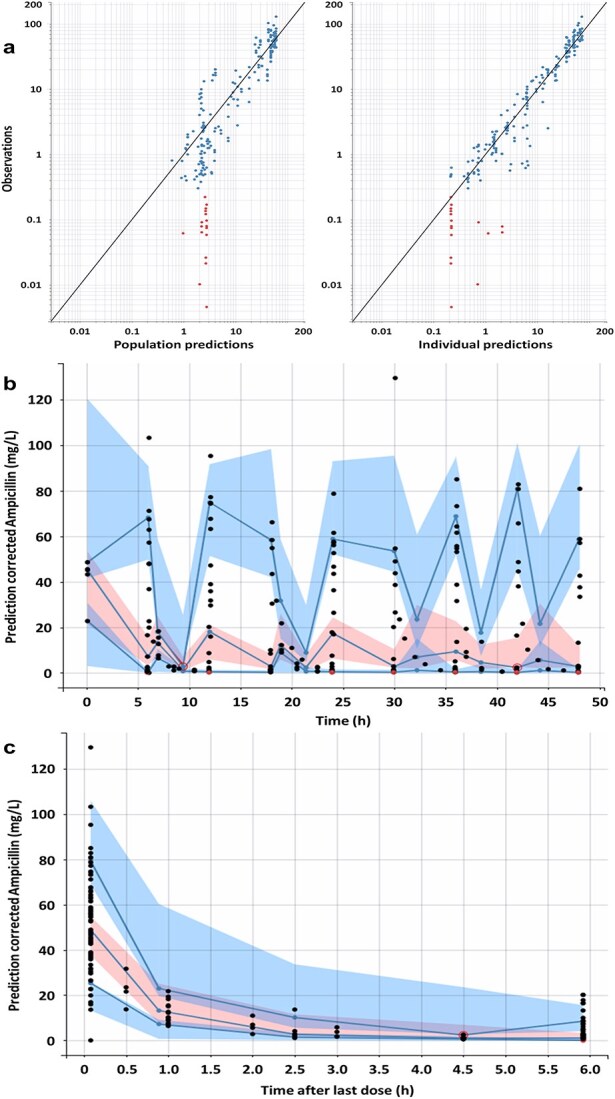
(a) Goodness-of-fit plots on a log–log scale: observations vs population predictions (left) and individual predictions (right) for AMP concentrations. The black line is the identity line, the blue dots are the observed values and the red dots are the censored values (below the quantification limit). (b) Prediction-corrected visual predictive checks for AMP concentrations of the final model represented as prediction corrected AMP concentrations over the time during the whole 48-h study period. Blue lines represent the 10th, 50th, and 90th percentiles of the observed concentrations, blue areas are the prediction intervals of the 10th and 90th percentiles, light red areas are the prediction intervals of the median, dark red area and red open circle are outliers, and the black and red dots are the observed and censored values, respectively. (c) Prediction-corrected visual predictive checks for AMP concentrations of the final model represented as prediction corrected AMP concentrations over the time within 6-h interdose intervals. Blue lines represent the 10th, 50th, and 90th percentiles of the observed concentrations, blue areas are the prediction intervals of the 10th and 90th percentiles, light red area is the prediction interval of the median, dark red area and red open circle are outliers, and the black and red dots are the observed and censored values, respectively. Abbreviation: AMP = ampicillin.

In addition, different error models were tested (constant, proportional, combined 1, and combined 2) and the error of the combined 1 model gave the best description of the data (ΔBIC = +431.43 for constant, ΔBIC = +7.53 for proportional, and ΔBIC = + 2.33 for combined 2) (Table S2). Finally, no significant association was observed between the estimates. The correlation between observed and predicted population and individual ampicillin concentrations (derived from the empirical Bayes estimate) is presented in Figure 1a. All the population PK parameters obtained from the final model are summarized in Table 2 and showed RSE values below 44.8% and no shrinkage (<6.3%).

#### Internal validation of the PK model

**Figure 2:**
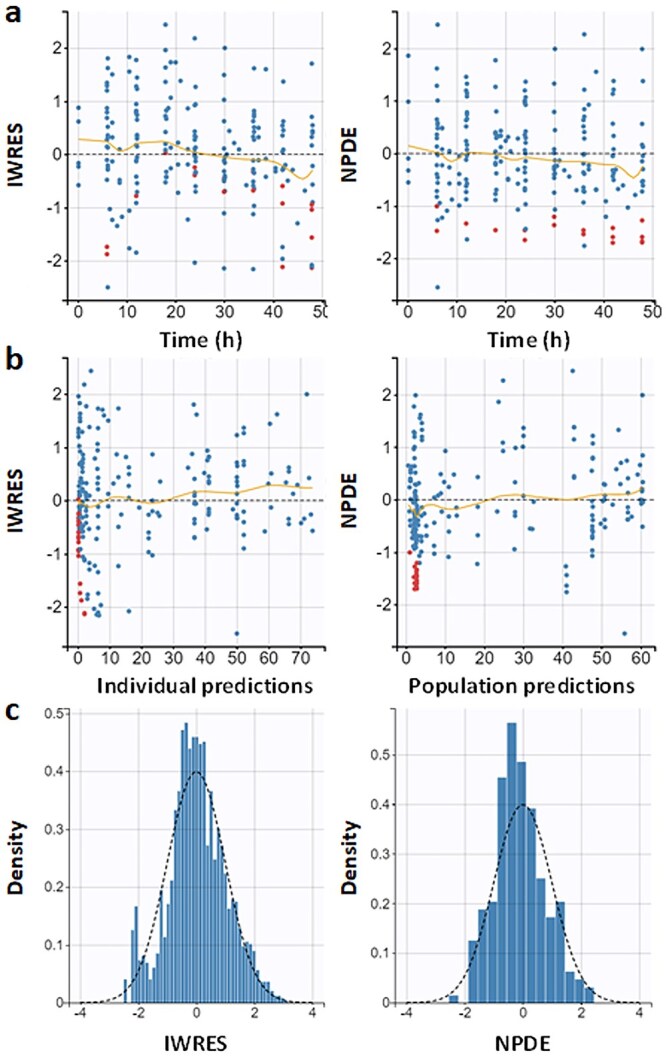
(a) Diagnostic plots: IWRES (left) and NPDE (right) as a function of time. The blue dots are the observed values, and the red dots are the censored values. (b) IWRES (left) and NPDE (right) as a function of individual and population predictions of ampicillin plasma concentrations. The blue dots are the observed values, and the red dots are the censored values. (c) Empirical probability density function is represented by blue columns and theoretical probability density function by the dashed black line of IWRES (left) and NPDE (right). Abbreviations: IWRES = individual weighted residuals; NPDE = normalized prediction distribution error.

The visual predictive check for the PK model of ampicillin plasma concentration showed that the 10th, 50th, and 90th percentiles of the observed data fell within the 90% prediction intervals obtained from model simulations (Figure 1b and c). Ninety-seven percent of the observed values were within the prediction intervals, confirming the ability of the model to describe the observed data. The distributions of the NPDE and IWRES metrics as a function of time or plasma ampicillin concentration (Figure 2) were symmetrical around zero value (symmetry test: *P* = .12 and *P* = .39, respectively) and normally distributed (Shapiro–Wilk test: *P* = .17 and *P* = .19, respectively). The parametric bootstrap procedure showed a convergence of 100% of the 1000 runs. The parameter based on the calculated 95% CI and bootstrap results supported the robustness and the stability of the final model (Table 2).

#### Foal’s minimum inhibitory concentrations

The bacterial species isolated from the 7 patients included *Clostridium perfringens* (from peritoneal fluid), *Streptococcus equi* subsp. *zooepidemicus* (isolated from 4 patients: 1 from blood and 3 from umbilical remnants), *Bacillus licheniformis* (from blood), and *Enterococcus faecalis* (from umbilical remnants). The MIC data obtained were compared with the ECOFF values outlined in the European Committee on Antimicrobial Susceptibility Testing (EUCAST) guidelines.^42^ When these values were not available, we used clinical breakpoints defined by CLSI.^43^ For *C perfringens*, specific EUCAST breakpoints for ampicillin are not available but the measured MIC of 0.06 μg/mL falls within the susceptible (S) range of 0.5 mg/L, given by CLSI for anaerobes (CLSI, 2025). For *S equi* subsp. *zooepidemicus*, the measured MIC was 0.06 mg/L, which also falls within the susceptible (S) range, as the measured MIC is well below the susceptibility EUCAST breakpoint (≤0.125) mg/L), indicating that the isolate is likely a wild-type strain. For *B licheniformis*, the measured MIC was 0.25 mg/L. Since EUCAST breakpoint of *Bacillus* spp. for ampicillin is not available, we used CLSI breakpoints (CLSI M45, 2016) that indicates ≤ 0.25 mg/L as breakpoint of susceptibility. For *E faecalis*, the measured MIC was 0.5 mg/L, which falls within the susceptible (S) range of EUCAST that is ≤ 4 mg/L, indicating that the isolate is a wild-type strain without acquired resistance.

#### Simulations of dosing regimens

After identifying the final model, simulations were conducted to evaluate the PTA for various ampicillin dosing regimens (15, 20, 30, and 40 mg/kg) administered at different intervals (q4h, q6h, and q8h) over a 48-h treatment period. The 3 PK/PD targets assessed were 50% fT > 1 × MIC, 50% fT > 4 × MIC, and 100% fT > 1 × MIC, with MICs ranging from 0.016 to 4 mg/L.

**Figure 3:**
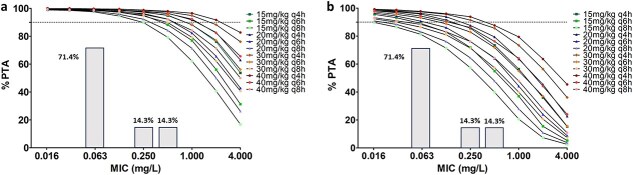
PTA of ampicillin vs MIC after intravenous bolus administration at different tested dosing regimens tested with the PK/PD target of 50% fT > 1 × MIC (a) and 100% fT > 1 × MIC (b). PTA is expressed as a percentage and the dashed line corresponds to 90% PTA. Columns represent the MIC distribution from the study population measured in 7 foals: *Bacillus licheniformis* (*n* = 1 with MIC = 0.25), *Streptococcus equi* subsp. *zooepidemicus* (*n* = 4 with MIC = 0.06), *Clostridium perfringens* (*n* = 1 with MIC = 0.06), and *Enterococcus faecalis* (*n* = 1 with MIC = 0.5). Abbreviations: MIC = minimum inhibitory concentration; PD = pharmacodynamics; PK = pharmacokinetics; PTA = probability of target attainment.

Figure 3 presents the PTA vs MIC profiles for the evaluated dosing regimens, specifically for PK/PD targets of 50% fT > MIC and 100% fT > MIC. In our study population, the 20 mg/kg q6h regimen achieved a PTA greater than 90% for MICs up to 0.5 mg/L when the PK/PD target was 50% fT > MIC. For the stricter target of 100% fT > MIC, however, the same dosing regimen (20 mg/kg q6h) resulted in a PTA greater than 90% for MICs up to 0.06 mg/L.

For the 100% fT > 1 × MIC target, only the q4h dosing interval—regardless of dose—achieved a PTA greater than 90% at an MIC of 0.125 mg/L. No tested regimen reached this PTA threshold for MICs above 0.25 mg/L. In contrast, for the 50% fT > 1 × MIC target, all tested regimens achieved a PTA greater than 90% at MICs ≤ 0.125 mg/L. At an MIC below 0.5 mg/L, the q4h and q6h regimens maintained a PTA of 90%. Finally, at an MIC of 2 mg/L, only the higher-dose regimen of 40 mg/kg q4h achieved a PTA greater than 90%.

**Figure 4:**
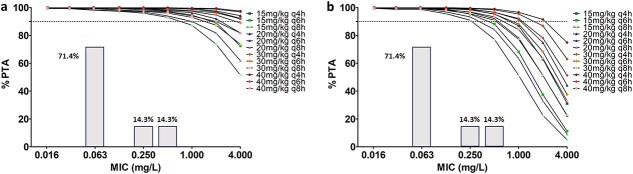
PTA of ampicillin vs MIC after intravenous bolus administration at different tested dosing regimens tested with the PK/PD target of 50% fT > 1 × MIC in the study population with a fixed median age of 10 h and a log-scale standard deviation of 0.5 (ie, approximately [6.07-16.49] in the natural scale) (a) and with a fixed median age of 120 h and a log-scale standard deviation of 0.5 (ie, approximately [72.8-197.8] in the natural scale) (b). PTA is expressed as a percentage and the dashed line corresponds to 90% PTA. Columns represent the MIC distribution from the study population measured in 7 foals: *Bacillus licheniformis* (*n* = 1 with MIC = 0.25), *Streptococcus equi* subsp. *zooepidemicus* (*n* = 4 with MIC = 0.06), *Clostridium perfringens* (*n* = 1 with MIC = 0.06), and *Enterococcus faecalis* (*n* = 1 with MIC = 0.5). Abbreviations: MIC = minimum inhibitory concentration; PD = pharmacodynamics; PK = pharmacokinetics; PTA = probability of target attainment.

Since age was identified as a covariate of ampicillin clearance and peripheral compartment volume (V2), the dosing regimens tested on our study population (with a mean age of 92.8 ± 75.8 h) were also evaluated in simulated populations with varying age distributions (Figure 4). Two age distributions with respective median ages of 10 and 120 h were considered. A PTA greater than 90% was observed for all tested regimens at MIC values up to 0.5 mg/L in the youngest simulated population and up to 0.25 mg/L in the oldest. In the youngest simulated population, all regimens, regardless of the dose administered every 4 h, were associated with a PTA above 90% up to a MIC of 4 mg/L. In contrast, in the oldest simulated population, only the high-dose regimen of 40 mg/kg every 4 h achieved a PTA greater than 90% up to a MIC of 2 mg/L.

## Discussion

The present study addresses existing knowledge gaps by proposing a validated population PK model of ampicillin in sick foals. The outcomes highlight the importance of age as covariate in the model, proving the importance of correcting dosing regimens based on foals characteristics, especially in ill subjects. The recommended dosage of ampicillin for foals (20-30 mg/kg of body weight per day, administered in 2 doses 12 h apart), as stated in the summary of product characteristics,^44^ was not followed. Instead, a dosage of 20 mg/kg every 6 h commonly recommended in the literature for treating hospitalized foals was chosen.^4^^,^^6^^,^^45^^,^^46^

The administered dosage of 20 mg/kg every 6 h achieved optimal PTA (≥90%) for the 7 MICs measured (ranging from 0.06 to 0.5 mg/L) with a 50% fT > 1 × MIC target.

However, PD simulations suggested that this regimen was inadequate for higher MICs or for achieving a 100% fT > 1 × MIC target. In such cases, higher doses (30-40 mg/kg) and more frequent dosing intervals (every 4 h) were required to maintain effective drug exposure. Age influenced PTA, with younger populations (median age of 10 h) achieving ≥ 90% PTA at higher MICs compared to older populations (median age of 120 h), where only the highest dose (40 mg/kg q4h) maintained ≥ 90% PTA at a MIC of 2 mg/L. This may be in line with an increase in volume of distribution and more mature renal function by 4 days of age, as measured by clearance studies in foals.^47^

Although no population PK studies in horses for ampicillin have been identified, a 2-compartment model for amoxicillin,^48^ a closely related antibiotic, has been described, providing a valuable framework for understanding the PKs of ampicillin in this species. The PK parameters of ampicillin in horses, as reported in the literature, show variability in clearance (Cl) and volume of distribution (Vd). Kondampati et al.^38^ reported a Cl of 0.33 ± 0.01 L/h/kg and a Vd of 0.44 ± 0.01 L/kg, which are relatively similar to the values reported by Winther et al.,^39^ where Cl was 0.42 ± 0.13 L/h/kg and Vd was 0.45 ± 0.05 L/kg. In contrast, van den Hoven et al.^41^ reported lower Cl (0.24 ± 0.02 L/h/kg) and a significantly larger Vd (0.68 ± 0.22 L/kg), possibly reflecting differences in dosing regimens and measurement techniques. In foals, Vd for water-soluble drugs is reportedly higher than in mature horses due to higher extracellular fluid volume.^49^ These variations highlight the influence of study design, administration routes, and physiological factors on the PKs of ampicillin in horses.

Following the selection of the 2-compartment model for ampicillin, the influence of covariates such as age, TBW, serum albumin, and serum creatinine on the PK parameters was further explored by integrating these factors into the basic model. In the present study, age was identified as a covariate for both peripheral compartment volume and ampicillin clearance, likely reflecting differences in maturation among the foals, whose ages ranged from 9 to 192 h. For clearance, serum creatinine showed a stronger association than age (Figure S1), which is expected given ampicillin’s primarily renal elimination, similar to other renally excreted antibiotics.^41^^,^^50^ In our study, we have highlighted a significant association between serum creatinine and ampicillin clearance. However, the presence of a markedly elevated serum creatinine value (63.8 mg/L) in one foal (the youngest) compared to the rest of the cohort (median: 12.8 [9.5-14.7] mg/L) limits the robustness of this association confirmed by bootstrapping. Thus, we chose to include age rather than serum creatinine in the PK model. Indeed, the elevated serum creatinine level observed in the youngest foal may represent spurious hypercreatininemia, a phenomenon that can be encountered in foals less than 72 h old.^49^ For informative purposes, we examined the impact of renal impairment on ampicillin PTA by evaluating serum creatinine levels (50, 100, and 150 mg/L), which are indicative of risk, injury, or acute renal failure, as previously described^51–53^ (Figure S2). As expected, higher PTA values were observed with worsening renal function for the same ampicillin doses. For the peripheral compartment V2, the influence of age on V2 may be related to weight gain and the developmental changes that occur as foals mature. These changes can alter the volume of distribution as the animal’s body composition and physiological characteristics evolve with age.^54–56^ However, the effect of physiological changes in Vd is considered more relevant for concentration-dependent drugs (eg, aminoglycosides) than time-dependent (eg, ampicillin) drugs.^49^^,^^57^ In addition, other factors affecting the fluid balance, such as volume contraction due to illness or expansion due to intravenous fluid therapy, should also be taken into consideration. Most foals experienced some degree of fluid loss, either dehydration or hypovolemia, and received isotonic crystalloids according to estimated deficits. The potential impact of these factors on ampicillin PK was not quantified in this population but should be considered in future analyses.

In the PK simulation, a fixed protein binding value of 15% was employed to estimate the unbound (free) concentration of ampicillin, as the pharmacological activity of antibiotics is primarily determined by the free drug concentration at the site of infection.^58^ The selection of this 15% binding value was based on the average protein binding percentages reported in the literature for horses. Notably, Dürr^37^ documented a protein binding of 8% for both penicillin G and ampicillin, while Kondampati et al.^41^ observed a binding of 14.5% for ampicillin in Indian Thoroughbred horses. In addition, Winther et al.^39^ reported a range of protein binding values for ampicillin in plasma, from 11.5% to 25.5%, depending on the drug concentration, with no significant concentration-dependent effect. The chosen 15% binding value is in alignment with existing data and provides a reliable estimate of the unbound ampicillin concentration, which is critical for accurately assessing its PK and PD properties in horses.^58^ None of the sampled foals exhibited hypoalbuminemia; thus, a relevant effect of hypoproteinemia on ampicillin distribution is unlikely. Although minor effects on protein binding from other factors, such as coadministered drugs, cannot be entirely excluded, these are considered negligible within the context of this preliminary PK modeling study and do not affect the overall conclusions, while remaining primarily relevant for future applications in therapeutic drug monitoring.

The tested doses of 15 and 20 mg/kg administered every 6-8 h were based on the dosing recommendations provided by Prescott and Baggot (2025).^59^ In addition, we included dosing regimens of 15 and 20 mg/kg every 4 h to evaluate the impact of more frequent supplemental doses.^60^ Higher doses of 40 and 30 mg/kg were also tested, as they correspond to the maximum recommended dose of 12 g found in the Standardized Compendium of Pharmaceuticals for human use of ampicillin.^61^ These dosing regimens were chosen to assess the PK and PD effects of varying dosages in horses, as described by Horspool et al.^32^

In critically ill foals, targeting 50% fT > 4 × MIC reflects a more aggressive PK/PD goal than traditional 50% fT > MIC, ensuring not only coverage but also maximizing bactericidal potential. The adoption of this approach is justified by the altered PK profiles observed in this population, the challenges associated with pathogen eradication, and the evidence linking higher antimicrobial exposures with improved clinical outcomes. Although a dose of 40 mg/kg q4h appears sufficient to achieve an acceptable PTA, continuous infusion may be a viable alternative, helping to maintain PTA while reducing overall ampicillin exposure and potentially lowering the risk of toxicity (eg, renal toxicity).

At 40 mg/kg, total ampicillin concentrations reach a *C*max equal to or greater than 100 mg/L, which is quite high and can increase the risk of toxic effects described for other antibiotics such as amoxicillin at high doses (150 mg/kg/day).^62–64^ The 100% fT is employed based on the theoretical antibacterial activity of penicillins, which are time-dependent antibiotics, whereas the 50% fT > MIC is grounded on the PK/PD target typically associated with ampicillin.^65–67^ We also tested the % fT > 1 × MIC or 4 × MIC, as numerous studies have demonstrated that exposure to β-lactam % fT > MIC is often suboptimal in a wide range of disease states and clinical settings.^68–70^

In this study, we considered a range of MICs for various bacterial pathogens relevant to equine infections.^23^ The tested MIC values were based on data from several sources, including studies by Léon et al.^71^ and van den Hoven et al.,^41^ which provided MIC values for common equine pathogens such as *Streptococcus* spp., *Escherichia coli*, and *Staphylococcus aureus*. In these studies, the MIC for *S aureus* was set at 2 mg/L, while for *S equi* subsp. *zooepidemicus*, a lower MIC range of 0.03-0.12 mg/L was reported.^39^ The clinical relevance of these MIC values is further underlined by the prevalence of resistant strains, with *E coli* showing fluctuating resistance patterns to penicillins between 2016 and 2019.^71^

Furthermore, based on the dosing regimen simulation conducted here, considering MICs ranging from 0.016 to 4 mg/L, ampicillin may not be an appropriate antibiotic for pathogens such as *E coli* or *E faecalis*, due to their EUCAST susceptibility breakpoints of 8 and 4 mg/L, respectively. For clarity and practical guidance, the key clinical take-home messages on ampicillin dosing regimens in foals, based on our study, are summarized in Table 3.

**Table 3:** Suggested ampicillin sodium dosing regimens in foals according to age and MIC values.

Clinical scenario	Recommended regimen
**Newborn foals (<72 h old) and MIC ≤ 0.5 mg/L**	20 mg/kg IV every 6 h generally adequate
**Older foals with MIC > 0.5 mg/L**	30-40 mg/kg IV every 4 h
**Any age—need to optimize PTA while limiting overall exposure**	Continuous infusion may be considered
**MIC > 4 mg/L (E coli, *E faecalis*)**	Switching to an alternative antimicrobial should be considered

Abbreviations: MIC, minimum inhibitory concentration; PTA, probability of target attainment.

In general, the variation in MIC values across different pathogens and over time reflects the complex antimicrobial susceptibility profiles encountered in veterinary medicine and the importance of continuous monitoring for effective treatment strategies.

This study has some limitations that should be considered when interpreting the results. The small sample size may restrict the generalizability of the findings. In addition, the analysis measured total ampicillin concentrations without distinguishing between bound and unbound drug fractions, which could impact PK interpretations. In addition, effects of concurrent therapies on drugs distribution, metabolism, and elimination were not insighted. Further research with a larger sample size and real-time assessment of drug stability is needed to validate these results.

In conclusion, the present study provides valuable insights into the use of ampicillin in neonatal healthcare, suggesting that an optimal PTA (≥90%) with a 50% fT > 1 × MIC can be achieved with a dosage of 20 mg/kg every 6 h for MIC below 0.5 mg/L. This approach enables more precise dosing for critically ill neonates, improving clinical outcomes and helping to mitigate the spread of antimicrobial resistance. Finally, the dosing regimens proposed in this study should be tested in further clinical trials.

## Supplementary Material

Figure_S1_aalag021

Figure_S2_aalag021

Tabl_S1_aalag021

Tabl_S2_aalag021

Tabl_S2_aalag021

Supplementary_Figures_captions_aalag021

## Off-label antimicrobial declaration

The authors declare no off-label use of antimicrobials.

## Institutional animal care and use committee or other approval declaration

Approved by the Animal Welfare Committee of the University of Bologna (Protocol No. 358467, ID No. 4626).

## Abbreviations

BICc: corrected Bayesian information criterion
BSV: between-subject variability
CLSI: Clinical and Laboratory Standards Institution
GOF: goodness-of-fit
IWRES: individual weighted residuals
MIC: minimum inhibitory concentration
PD: pharmacodynamics
PK: pharmacokinetics
PTA: probability of target attainment
SAEM: stochastic approximation expectation–maximization
TBW: total body weight

## References

[1] Perkins GA, Wagner B. The development of equine immunity: current knowledge on immunology in the young horse. Equine Vet J. 2015;47:267–274. 10.1111/evj.12387 25405920

[2] Dunkel B, Johns IC. Antimicrobial use in critically ill horses. J Vet Emerg Crit Care (San Antonio). 2015;25:89–100. 10.1111/vec.12275 25582245

[3] Fielding CL, Magdesian KG. Sepsis and septic shock in the equine neonate. Vet Clin North Am Equine Pract. 2015;31:483–496. 10.1016/j.cveq.2015.09.001 26612744

[4] Floyd EF, Easton-Jones CA, Theelen MJP. Systemic antimicrobial therapy in foals. Equine Vet Educ. 2022;34:49–56. 10.1111/eve.13467

[5] Theelen MJP, Wilson WD, Byrne BA, Edman JM, Kass PH, Magdesian KG. Initial antimicrobial treatment of foals with sepsis: do our choices make a difference? Vet J. 2019;243:74–76. 10.1016/j.tvjl.2018.11.012 30606442

[6] Magdesian KG . Antimicrobial pharmacology for the neonatal foal. Vet Clin North Am Equine Pract. 2017;33:47–65. 10.1016/j.cveq.2016.12.004 28325182

[7] Monaghan K, Labato M, Papich M. Ampicillin pharmacokinetics in azotemic and healthy dogs. J Vet Intern Med. 2021;35:987–992. 10.1111/jvim.16026 33474795 PMC7995374

[8] Proceedings-62nd-Annual-Convention-2016.pdf. American Association of Equine Practitioners. Accessed April 12, 2025. https://aaep.org/wp-content/uploads/2024/02/Proceedings-62nd-Annual-Convention-2016.pdf

[9] Onufrak NJ, Forrest A, Gonzalez D. Pharmacokinetic and pharmacodynamic principles of anti-infective dosing. Clin Ther. 2016;38:1930–1947. 10.1016/j.clinthera.2016.06.015 27449411 PMC5039113

[10] Levison ME, Levison JH. Pharmacokinetics and pharmacodynamics of antibacterial agents. Infect Dis Clin North Am. 2009;23:791–vii. 10.1016/j.idc.2009.06.008 19909885 PMC3675903

[11] Baggot JD . Drug therapy in the neonatal foal. Vet Clin North Am Equine Pract. 1994;10:87–107. 10.1016/s0749-0739(17)30370-x 8039037

[12] Loberg JJ . Foal physiology and special considerations during anesthesia. Veterinary Technician. Accessed February 9, 2026. https://vetfolio-vetstreet.s3.amazonaws.com/mmah/68/cc43218dcd493cb07fd2ee7b25296a/fileVT0210_CE_Loberg.pdf

[13] Knych HK, Steffey EP, Mitchell MM, Casbeer HC. Effects of age on the pharmacokinetics and selected pharmacodynamics of intravenously administered fentanyl in foals. Equine Vet J. 2015;47:72–77. 10.1111/evj.12246 25263971

[14] Caprile KA, Short CR. Pharmacologic considerations in drug therapy in foals. Vet Clin North Am Equine Pract. 1987;3:123–144. 10.1016/s0749-0739(17)30694-6 3555723

[15] Papich MG . Pharmacokinetic–pharmacodynamic (PK–PD) modeling and the rational selection of dosage regimes for the prudent use of antimicrobial drugs. Vet Microbiol. 2014;171:480–486. 10.1016/j.vetmic.2013.12.021 24513278

[16] Rao GG, Landersdorfer CB. Antibiotic pharmacokinetic/pharmacodynamic modelling: MIC, pharmacodynamic indices and beyond. Int J Antimicrob Agents. 2021;58:106368. 10.1016/j.ijantimicag.2021.106368 34058336

[17] Minichmayr IK, Aranzana-Climent V, Friberg LE. Pharmacokinetic/pharmacodynamic models for time courses of antibiotic effects. Int J Antimicrob Agents. 2022;60:106616. 10.1016/j.ijantimicag.2022.106616 35691605

[18] Wicha SG, Märtson A, Nielsen EI, et al. From therapeutic drug monitoring to model-informed precision dosing for antibiotics. Clin Pharmacol Ther. 2021;109:928–941. 10.1002/cpt.2202 33565627

[19] Ahmad I, Huang L, Hao H, Sanders P, Yuan Z. Application of PK/PD modeling in veterinary field: dose optimization and drug resistance prediction. Biomed Res Int. 2016;2016:5465678. 10.1155/2016/5465678 26989688 PMC4771886

[20] Luo W, Chen D, Wu M, et al. Pharmacokinetics/pharmacodynamics models of veterinary antimicrobial agents. J Vet Sci. 2019;20:e40. 10.4142/jvs.2019.20.e40 31565887 PMC6769327

[21] Toutain PL . Pharmacokinetic/pharmacodynamic integration in drug development and dosage-regimen optimization for veterinary medicine. AAPS J. 2015;4:38. 10.1208/ps040438 PMC2751327 12646010

[22] Gao T, Liu X, Qiu D, et al. Ex vivo pharmacokinetic/pharmacodynamic integration model of cefquinome against *Escherichia coli* in foals. Vet Sci. 2025;12:294. 10.3390/vetsci12040294 40284796 PMC12031376

[23] VETAMPLIUS 10 g/50 ml Powder and solvent for solution for injection | UPD. Accessed January 22, 2025. https://medicines.health.europa.eu/veterinary/en/600000093465

[24] Del Prete C, Lanci A, Cocchia N, et al. Venous blood gas parameters, electrolytes, glucose and lactate concentration in sick neonatal foals: direct venipuncture versus push-pull technique. Equine Vet J. 2021;53:488–494. 10.1111/evj.13332 32770680

[25] Clinical and Laboratory Standards Institute (CLSI) . CLSI Methods for Dilution Antimicrobial Susceptibility Tests for Bacteria that Grow Aerobically. 11th ed. CLSI; 2018.

[26] Clinical and Laboratory Standards Institute (CLSI) . CLSI Methods for Antimicrobial Susceptibility Testing of Anaerobic Bacteria. 9th ed. CLSI; 2018.

[27] Bardhi A, Lanci A, Mannini A, Castagnetti C, Barbarossa A. A laboratory protocol for routine therapeutic drug monitoring of beta-lactams antimicrobials in horses and dogs. Antibiotics. 2025;14:390. 10.3390/antibiotics14040390 40298550 PMC12024143

[28] Cazaubon Y, Talineau Y, Feliu C, et al. Population pharmacokinetics modelling and simulation of mitotane in patients with adrenocortical carcinoma: an individualized dose regimen to target all patients at three months? Pharmaceutics. 2019;11:566. 10.3390/pharmaceutics11110566 31683663 PMC6920765

[29] Cazaubon Y, Mauprivez C, Feliu C, et al. Population pharmacokinetics of articaine with 1:200,000 epinephrine during third molar surgery and simulation of high-dose regimens. Eur J Pharm Sci. 2018;114:38–45. 10.1016/j.ejps.2017.11.027 29197630

[30] Konecki C, Holm M, Djerada Z. Negative impact of ST-segment elevation myocardial infarction and morphine dose on ticagrelor uptake and pharmacodynamics: a population PK/PD analysis of pooled individual participant data. Clin Pharmacokinet. 2023;62:905–920. 10.1007/s40262-023-01243-5 37097605

[31] Sarasola P, Horspool LJ, McKellar QA. Effect of changes in urine pH on plasma pharmacokinetic variables of ampicillin sodium in horses. Am J Vet Res. 1992;53:711–715. 10.2460/ajvr.1992.53.05.711 1326242

[32] Horspool LJ, Sarasola P, McKellar QA. Disposition of ampicillin sodium in horses, ponies and donkeys after intravenous administration. Equine Vet J Suppl. 1992;11:59–61. 10.1111/j.2042-3306.1992.tb04775.x 9109963

[33] Djerada Z, Feliu C, Cazaubon Y, et al. Population pharmacokinetic-pharmacodynamic modeling of ropivacaine in spinal anesthesia. Clin Pharmacokinet. 2018;57:1135–1147. 10.1007/s40262-017-0617-2 29236228

[34] Allard Q, Djerada Z, Pouplard C, et al. Real life population pharmacokinetics modelling of eight factors VIII in patients with severe haemophilia A: is it always relevant to switch to an extended half-life? Pharmaceutics. 2020;12:380. 10.3390/pharmaceutics12040380 32326156 PMC7238177

[35] Beltrand J, Baptiste A, Busiah K, et al. Glibenclamide oral suspension: suitable and effective in patients with neonatal diabetes. Pediatr Diabetes. 2019;20:246–254. 10.1111/pedi.12823 30684309

[36] Mould DR, Upton RN. Basic concepts in population modeling, simulation, and model-based drug development-part 2: introduction to pharmacokinetic modeling methods. CPT Pharmacometrics Syst Pharmacol. 2013;2:e38. 10.1038/psp.2013.14 23887688 PMC3636497

[37] Dürr A . Comparison of the pharmacokinetics of penicillin G and ampicillin in the horse. Res Vet Sci. 1976;20:24–29. 1257624

[38] Kondampati KD, Saini SPS, Sidhu PK, et al. Pharmacokinetic-pharmacodynamic study of ampicillin-cloxacillin combination in Indian thoroughbred horses (*Equus caballus*) and safety evaluation of the computed dosage regimen. J Equine Vet Sci. 2022;115:104020. 10.1016/j.jevs.2022.104020 35605881

[39] Winther L, Baptiste KE, Friis C. Pharmacokinetics in pulmonary epithelial lining fluid and plasma of ampicillin and pivampicillin administered to horses. Res Vet Sci. 2012;92:111–115. 10.1016/j.rvsc.2010.11.001 21144541

[40] Mouton JW, Brown DFJ, Apfalter P, et al. The role of pharmacokinetics/pharmacodynamics in setting clinical MIC breakpoints: the EUCAST approach. Clin Microbiol Infect. 2012;18:E37–E45. 10.1111/j.1469-0691.2011.03752.x 22264314

[41] van den Hoven R, Hierweck B, Dobretsberger M, Ensink JM, Meijer LA. Intramuscular dosing strategy for ampicillin sodium in horses, based on its distribution into tissue chambers before and after induction of inflammation. J Vet Pharmacol Ther. 2003;26:405–411. 10.1046/j.0140-7783.2003.00532.x 14962051

[42] eucast: EUCAST. Accessed March 5, 2025. https://www.eucast.org/

[43] v_15.0_Breakpoint_Tables.pdf. Accessed March 5, 2025. https://www.eucast.org/fileadmin/src/media/PDFs/EUCAST_files/Breakpoint_tables/v_15.0_Breakpoint_Tables.pdf

[44] SPC_131096.pdf. Accessed March 5, 2025. https://www.vmd.defra.gov.uk/productinformationdatabase/files/SPC_Documents/SPC_131096.PDF

[45] Antimicrobial therapy in neonatal foals. Giguere. Accessed January 21, 2025. https://colab.ws/articles/10.2746%2F095777309x445352

[46] Hardefeldt LY, Bailey KE, Slater J. Overview of the use of antimicrobial drugs for the treatment of bacterial infections in horses. Equine Vet Educ. 2021;33:602–611. 10.1111/eve.13371

[47] Brewer BD, Clement SF, Lotz WS, Gronwall R. A comparison of inulin, para-aminohippuric acid, and endogenous creatinine clearances as measures of renal function in neonatal foals. J Vet Intern Med. 1990;4:301–305. 10.1111/j.1939-1676.1990.tb03127.x 2074554

[48] Errecalde JO, Carmely D, Mariño EL, Mestorino N. Pharmacokinetics of amoxycillin in normal horses and horses with experimental arthritis. J Vet Pharmacol Ther. 2001;24:1–6. 10.1046/j.1365-2885.2001.00290.x 11348481

[49] Kami G, Merritt AM, Duelly P. Preliminary studies of plasma and extracellular fluid volume in neonatal ponies. Equine Vet J. 1984;16:356–358. 10.1111/j.2042-3306.1984.tb01942.x 6479132

[50] Martín-Jiménez T, Papich MG, Riviere JE. Population pharmacokinetics of gentamicin in horses. Am J Vet Res. 1998;59:1589–1598. 10.2460/ajvr.1998.59.12.1589 9858412

[51] Bellomo R, Ronco C, Kellum JA, Mehta RL, Palevsky P, Acute Dialysis Quality Initiative workgroup. Acute dialysis quality initiative workgroup. Acute renal failure—definition, outcome measures, animal models, fluid therapy and information technology needs: the Second International Consensus Conference of the Acute Dialysis Quality Initiative (ADQI) Group. Crit Care. 2004;8:R204–R212. 10.1186/cc2872 15312219 PMC522841

[52] Chaney KP, Holcombe SJ, Schott HC, Barr BS. Spurious hypercreatininemia: 28 neonatal foals (2000-2008). J Vet Emerg Crit Care (San Antonio). 2010;20:244–249. 10.1111/j.1476-4431.2010.00525.x 20487253

[53] Geor RJ . Acute renal failure in horses. Vet Clin North Am Equine Pract. 2007;23:577–591, v-vi. 10.1016/j.cveq.2007.09.007 18061851

[54] Swain E, Magdesian K, Kass P, Edman J, Knych H. Pharmacokinetics of metronidazole in foals: influence of age within the neonatal period. J Vet Pharmacol Ther. 2014;38:227–234. 10.1111/jvp.12164 25271172

[55] Norman WM, Court MH, Greenblatt DJ. Age-related changes in the pharmacokinetic disposition of diazepam in foals. Am J Vet Res. 1997;58:878–880. 10.2460/ajvr.1997.58.08.878 9256974

[56] Burton AJ, Giguère S, Warner L, Alhamhoom Y, Arnold RD. Effect of age on the pharmacokinetics of a single daily dose of gentamicin sulfate in healthy foals. Equine Vet J. 2013;45:507–511. 10.1111/j.2042-3306.2012.00683.x 23173817

[57] Magdesian KG, Wilson WD, Mihalyi J. Pharmacokinetics of a high dose of amikacin administered at extended intervals to neonatal foals. Am J Vet Res. 2004;65:473–479. 10.2460/ajvr.2004.65.473 15077690

[58] Briscoe SE, McWhinney BC, Lipman J, Roberts JA, Ungerer JPJ. A method for determining the free (unbound) concentration of ten beta-lactam antibiotics in human plasma using high performance liquid chromatography with ultraviolet detection. J Chromatogr B Analyt Technol Biomed Life Sci. 2012;907:178–184. 10.1016/j.jchromb.2012.09.016 23026224

[59] Hardefelt LY, Prescott JF. Beta-lactam antibiotics: penam penicillins. In: Dowing PM, Prescott JF, Baptiste KE, eds. Antimicrohial Therapy in Veterinary Medicine. Blackwell Scientific Publications; 2025:121–142. 10.1002/9781119654629.ch7

[60] Davis J . Antimicrobial therapy in the neonatal foal. In: Wong DM, Wilkins PA, eds. Equine Neonatal Medicine. Wiley Blackwell; 2024:1328–1343 10.1002/9781119617228.ch61.

[61] Résumé des caractéristiques du produit—AMPICILLINE PANPHARMA 1 g, poudre et solution pour préparation injectable—Base de données publique des médicaments. Accessed January 21, 2025. https://base-donnees-publique.medicaments.gouv.fr/affichageDoc.php?specid=61392249&typedoc=R

[62] Guilhaumou R, Benaboud S, Bennis Y, et al. Optimization of the treatment with beta-lactam antibiotics in critically ill patients-guidelines from the French Society of Pharmacology and Therapeutics (Société Française de Pharmacologie et Thérapeutique-SFPT) and the French Society of Anaesthesia and Intensive Care Medicine (Société Française d’Anesthésie et Réanimation-SFAR). Crit Care. 2019;23:104. 10.1186/s13054-019-2378-9 30925922 PMC6441232

[63] Clément M, Anglade F, Gibold L, et al. Amoxicillin blood concentration in high-dose intravenous discontinuous amoxicillin: look beyond numbers. Max-Amox study. Clin Ther. 2024;47:212–218. 10.1016/j.clinthera.2024.12.003 39734108

[64] Demotier S, Limelette A, Charmillon A, et al. Incidence, associated factors, and effect on renal function of amoxicillin crystalluria in patients receiving high doses of intravenous amoxicillin (the CRISTAMOX study): a cohort study. EClinicalMedicine. 2022;45:101340. 10.1016/j.eclinm.2022.101340 35295665 PMC8919213

[65] Craig WA . Pharmacokinetic/pharmacodynamic parameters: rationale for antibacterial dosing of mice and men. Clin Infect Dis. 1998;26:1–10; quiz 11-12. 10.1086/516284 9455502

[66] Jacobs MR . How can we predict bacterial eradication? Int J Infect Dis. 2003;7:S13–S20. 10.1016/s1201-9712(03)90066-x 12839703

[67] Berry AV, Kuti JL. Pharmacodynamic thresholds for beta-lactam antibiotics: a story of mouse versus man. Front Pharmacol. 2022;13:833189. 10.3389/fphar.2022.833189 35370708 PMC8971958

[68] Fratoni AJ, Nicolau DP, Kuti JL. A guide to therapeutic drug monitoring of β-lactam antibiotics. Pharmacotherapy. 2021;41:220–233. 10.1002/phar.2505 33480024

[69] Udy AA, Varghese JM, Altukroni M, et al. Subtherapeutic initial β-lactam concentrations in select critically ill patients: association between augmented renal clearance and low trough drug concentrations. Chest. 2012;142:30–39. 10.1378/chest.11-1671 22194591

[70] Abdul-Aziz MH, Lipman J, Roberts JA. Identifying “at-risk” patients for sub-optimal beta-lactam exposure in critically ill patients with severe infections. Crit Care. 2017;21:283. 10.1186/s13054-017-1871-2 29157264 PMC5697074

[71] Léon A, Castagnet S, Maillard K, Paillot R, Giard JC. Evolution of in vitro antimicrobial susceptibility of equine clinical isolates in France between 2016 and 2019. Animals (Basel). 2020;10:812. 10.3390/ani10050812 32392891 PMC7278474

