# Supplementary figures and images for: A population pharmacokinetic study of ampicillin therapy in hospitalized foals
Source: J Vet Intern Med. 2026 Feb 23;40(1):aalag021. doi: 10.1093/jvimsj/aalag021 (PMC12927874; doi:10.1093/jvimsj/aalag021)

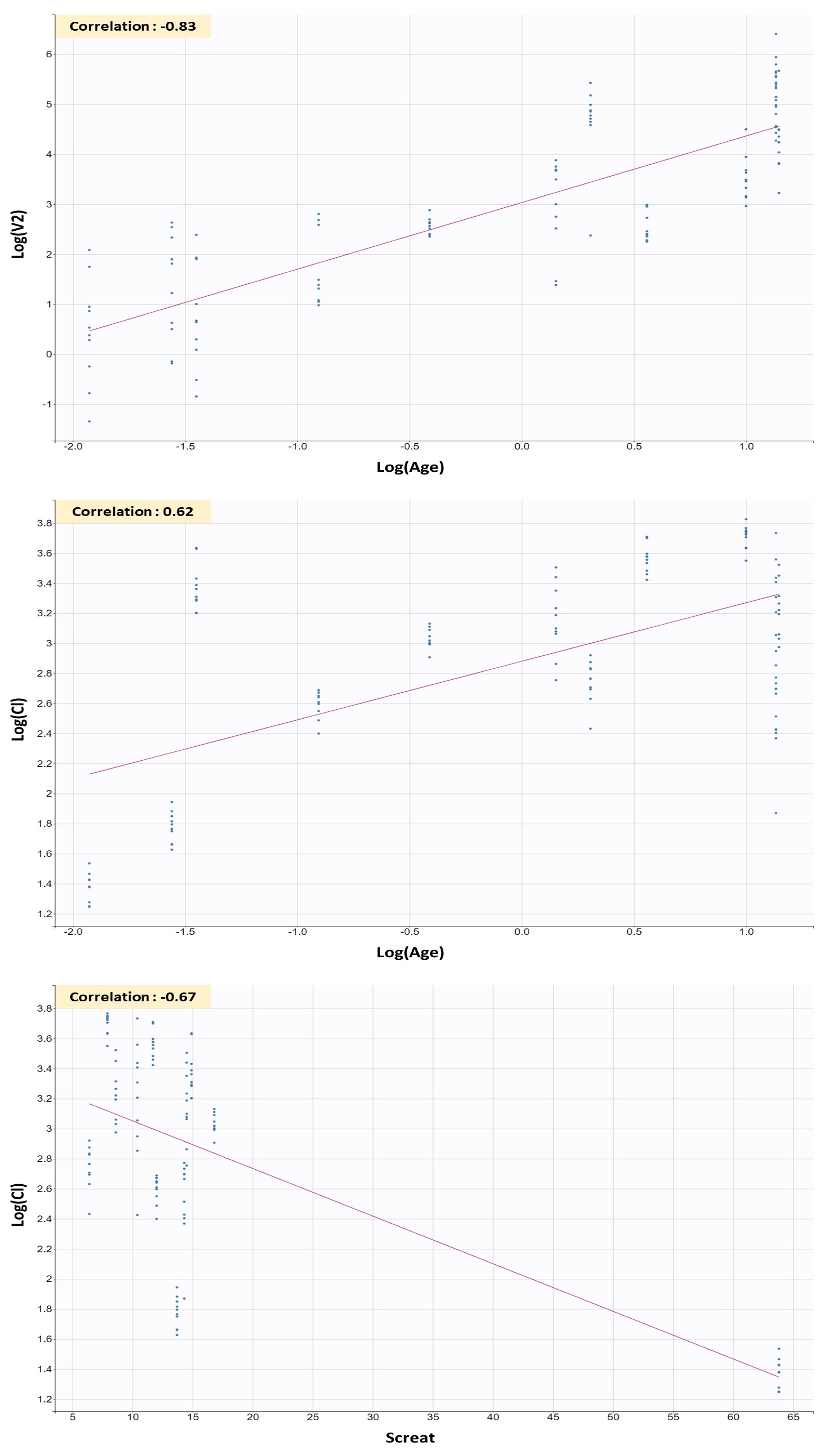

Supplement: Figure_S1_aalag021 [file figure_s1_aalag021.jpeg]

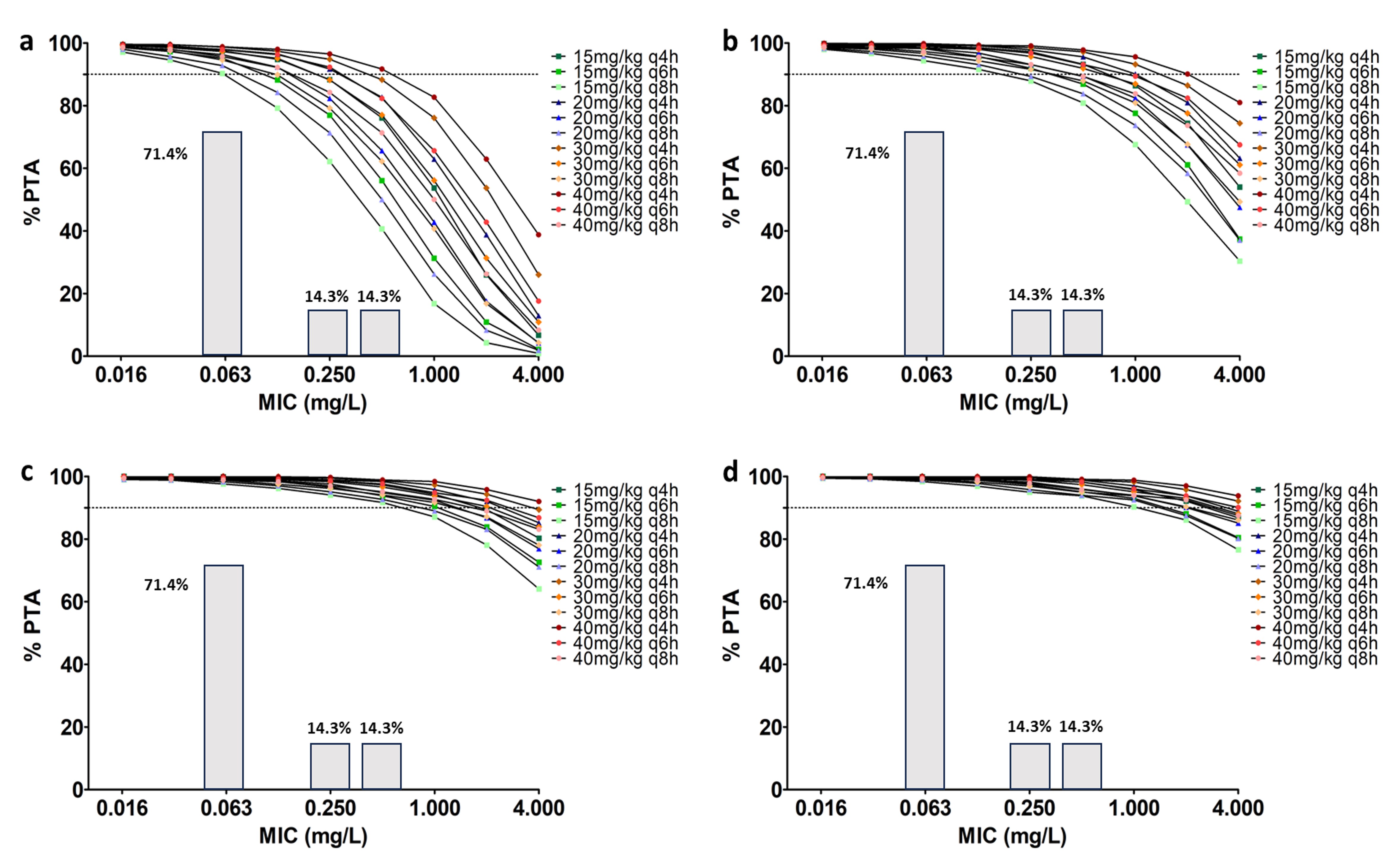

Supplement: Figure_S2_aalag021 [file figure_s2_aalag021.jpeg]
