# Supplementary material for: A population pharmacokinetic study of ampicillin therapy in hospitalized foals
Source: J Vet Intern Med. 2026 Feb 23;40(1):aalag021. doi: 10.1093/jvimsj/aalag021 (PMC12927874; doi:10.1093/jvimsj/aalag021)
Supplement: Tabl_S1_aalag021 [file tabl_s1_aalag021.docx]

**Table S1.** Schedule of blood sample collection timepoints over the first 48 hours of treatment for the 12 foals enrolled in the study (F1–F12).

| Time (h) | F1 | F2 | F3 | F4 | F5 | F6 | F7 | F8 | F9 | F10 | F11 | F12 |  |
| --- | --- | --- | --- | --- | --- | --- | --- | --- | --- | --- | --- | --- | --- |
| 0.08 |  |  |  |  |  |  | x | x | x | x | x | x |  |
| 5.92 | x | x | x | x | x | x | x | x | x | x | x | x |  |
| 6.08 | x | x | x | x | x | x |  | x | x | x | x | x |  |
| 6.50 | x |  |  |  |  |  |  |  |  |  |  |  |  |
| 7.00 | x | x | x | x | x |  |  |  |  |  |  |  |  |
| 8.00 | x |  |  |  |  |  |  |  |  |  |  |  |  |
| 8.50 |  | x | x | x |  |  |  |  |  |  |  |  |  |
| 9.00 | x |  |  |  |  |  |  |  |  |  |  |  |  |
| 10.50 | x | x | x | x |  |  |  |  |  |  |  |  |  |
| 11.92 | x | x | x | x | x | x | x | x | x | x | x | x |  |
| 12.08 | x | x | x | x | x | x | x | x | x | x | x | x |  |
| 17.92 | x | x | x | x | x |  | x |  | x | x | x | x |  |
| 18.08 | x | x | x | x | x |  |  |  |  |  |  |  |  |
| 18.50 | x |  |  |  |  |  |  |  |  |  |  |  |  |
| 19.00 | x | x | x | x |  |  |  |  |  |  |  |  |  |
| 20.00 | x |  |  |  |  |  |  |  |  |  |  |  |  |
| 20.50 |  | x | x | x |  |  |  |  |  |  |  |  |  |
| 21.00 | x |  |  |  |  |  |  |  |  |  |  |  |  |
| 22.50 | x | x | x | x |  |  |  |  |  |  |  |  |  |
| 23.92 | x | x | x | x | x | x | x | x |  | x | x | x |  |
| 24.08 | x | x | x | x | x | x | x | x |  | x | x | x |  |
| 29.92 | x | x | x | x | x | x | x | x |  | x | x | x |  |
| 30.08 | x | x | x | x | x | x |  |  |  |  |  |  |  |
| 30.50 | x |  |  |  |  |  |  |  |  |  |  |  |  |
| 31.00 | x |  |  |  |  |  |  |  |  |  |  |  |  |
| 32.00 | x |  |  |  |  |  |  |  |  |  |  |  |  |
| 33.00 | x |  |  |  |  |  |  |  |  |  |  |  |  |
| 34.50 | x |  |  |  |  |  |  |  |  |  |  |  |  |
| 35.92 | x | x | x | x | x |  | x | x |  | x | x | x |  |
| 36.08 | x | x | x | x | x |  | x | x |  | x | x | x |  |
| 37.00 |  | x | x | x |  |  |  |  |  |  |  |  |  |
| 38.50 |  | x | x | x |  |  |  |  |  |  |  |  |  |
| 40.50 |  | x |  | x |  |  |  |  |  |  |  |  |  |
| 41.92 | x | x | x |  | x | x | x | x |  |  | x | x |  |
| 42.08 | x | x | x | x | x | x |  |  |  |  |  |  |  |
| 42.50 | x |  |  |  |  |  |  |  |  |  |  |  |  |
| 43.00 | x |  |  |  |  |  |  |  |  |  |  |  |  |
| 44.00 | x |  |  |  |  |  |  |  |  |  |  |  | |
| 45.00 | x |  |  |  |  |  |  |  |  |  |  |  | |
| 46.50 | x |  |  |  |  |  |  |  |  |  |  |  | |
| 47.92 | x | x | x | x | x | x | x | x |  |  | x | x | |
| 48.08 | x | x | x | x | x | x |  |  |  |  |  |  | |
