# Supplementary material for: A population pharmacokinetic study of ampicillin therapy in hospitalized foals
Source: J Vet Intern Med. 2026 Feb 23;40(1):aalag021. doi: 10.1093/jvimsj/aalag021 (PMC12927874; doi:10.1093/jvimsj/aalag021)
Supplement: Tabl_S2_aalag021 [file tabl_s2_aalag021.docx]

**Table S2.** corrected Bayesian Information Criterion (BICc) values associated with each pharmacokinetic model studied for plasma ampicillin concentrations. The final selected model is in bold.

| **No. of compartments** | **Error Model** | **BICc** | **Comment** |
| --- | --- | --- | --- |
| 1 | Combined 1 | 1280.38 | - |
| 3 | Combined 1 | 1232.47 | - |
| **2** | Combined 1 | 1217.28 | - |
|  | Combined 1 | 1214.61 | ωQ (fixed) |
|  | Combined 1 | 1210.41 | ωQ (fixed); log(Age)/V2 |
|  | Combined 1 | 1204.88 | ωQ (fixed); log(Age)/V2; V2 (fixed) |
|  | **Combined 1** | **1202.40** | **ωQ (fixed); log(Age)/V2; V2 (fixed); log(Age)/Cl** |
|  | Combined 2 | 1204.73 |  |
|  | Constant | 1633.83 |  |
|  | Proportional | 1209.93 |  |

Cl and V2 are the clearance of ampicillin and the volume of the peripheral compartment, respectively; ωQ represents the standard deviation of the interindividual variability associated with the intercompartment clearance parameter Q; log(Age)/V2 and log(Age)/Cl correspond to adding log(Age) as a covariate to V2 and Cl, respectively. Constant error model: C_obs_ = C_pred_ + *a* × *ɛ*; Proportional error model: C_obs_ = C_pred_ + *b* × C_pred_ × *ɛ*; Combined error model 1: C_obs_ = C_pred_ + (*a* + *b* × C_pred_) × *ɛ*; Combined error model 2: C_obs_ = C_pred_ + (*a*^2^ + *b*^2^ × C_pred_^2^)^1/2^ × *ɛ* (combined 2), where C_obs_ is the observed concentration; C_pred_ is the predicted concentration, *a* is the additive residual error constant, *b* is the proportional residual error constant, and *ɛ* is the random error term.
