# Supplementary material for: A population pharmacokinetic study of ampicillin therapy in hospitalized foals
Source: J Vet Intern Med. 2026 Feb 23;40(1):aalag021. doi: 10.1093/jvimsj/aalag021 (PMC12927874; doi:10.1093/jvimsj/aalag021)
Supplement: Supplementary_Figures_captions_aalag021 [file supplementary_figures_captions_aalag021.docx]

**Figure S1.** Correlation between individual parameters (clearance (Cl) and peripheral compartment volume (V2)) and covariates (age and serum creatinine (Scr)). Blue dots are individual parameter estimates obtained from bootstrap replicates. The red line indicates the linear regression fit. The Pearson correlation coefficient for each parameter-covariate pair is displayed in the yellow box.

**Figure S2.** Probability of target attainment (PTA) of ampicillin versus MIC after intravenous bolus administration at different tested dosing regimens tested with the PK/PD target of 50% fT > 4×MIC. Simulations were conducted for a typical serum creatinine concentration of 13.2 mg/L with a log standard deviation of 0.57 (observed) (a), and for fixed serum creatinine levels of 50 mg/L (b), 100 mg/L (c), and 150 mg/L (d), all with the same standard deviation. PTA is expressed as a percentage and the dashed line corresponds to 90% PTA. Columns represent the MIC distribution from the study population measured in 7 foals: *Bacillus licheniformis* (n = 1 with MIC = 0.25), *Streptococcus equi* subsp. *zooepidemicus* (n = 4 with MIC = 0.06), *Clostridium perfringens* (n = 1 with MIC = 0.06) and *Enterococcus faecalis* (n = 1 with MIC = 0.5).
